# Patients’ Perspectives on the Data Confidentiality, Privacy, and Security of mHealth Apps: Systematic Review

**DOI:** 10.2196/50715

**Published:** 2024-05-31

**Authors:** Nasser Alhammad, Mohannad Alajlani, Alaa Abd-alrazaq, Gregory Epiphaniou, Theodoros Arvanitis

**Affiliations:** 1 Institute of Digital Healthcare, WMG University of Warwick Coventry United Kingdom; 2 Health Informatics Saudi Electronic University Jeddah Saudi Arabia; 3 AI Center for Precision Health Weill Cornell Medicine Doha Qatar; 4 School of Engineering University of Birmingham Birmingham United Kingdom

**Keywords:** mobile health apps, mHealth apps, mobile health, privacy, confidentiality, security, awareness, perspectives, mobile phone

## Abstract

**Background:**

Mobile health (mHealth) apps have the potential to enhance health care service delivery. However, concerns regarding patients’ confidentiality, privacy, and security consistently affect the adoption of mHealth apps. Despite this, no review has comprehensively summarized the findings of studies on this subject matter.

**Objective:**

This systematic review aims to investigate patients’ perspectives and awareness of the confidentiality, privacy, and security of the data collected through mHealth apps.

**Methods:**

Using the PRISMA (Preferred Reporting Items for Systematic Reviews and Meta-Analyses) guidelines, a comprehensive literature search was conducted in 3 electronic databases: PubMed, Ovid, and ScienceDirect. All the retrieved articles were screened according to specific inclusion criteria to select relevant articles published between 2014 and 2022.

**Results:**

A total of 33 articles exploring mHealth patients’ perspectives and awareness of data privacy, security, and confidentiality issues and the associated factors were included in this systematic review. Thematic analyses of the retrieved data led to the synthesis of 4 themes: concerns about data privacy, confidentiality, and security; awareness; facilitators and enablers; and associated factors. Patients showed discordant and concordant perspectives regarding data privacy, security, and confidentiality, as well as suggesting approaches to improve the use of mHealth apps (facilitators), such as protection of personal data, ensuring that health status or medical conditions are not mentioned, brief training or education on data security, and assuring data confidentiality and privacy. Similarly, awareness of the subject matter differed across the studies, suggesting the need to improve patients’ awareness of data security and privacy. Older patients, those with a history of experiencing data breaches, and those belonging to the higher-income class were more likely to raise concerns about the data security and privacy of mHealth apps. These concerns were not frequent among patients with higher satisfaction levels and those who perceived the data type to be less sensitive.

**Conclusions:**

Patients expressed diverse views on mHealth apps’ privacy, security, and confidentiality, with some of the issues raised affecting technology use. These findings may assist mHealth app developers and other stakeholders in improving patients’ awareness and adjusting current privacy and security features in mHealth apps to enhance their adoption and use.

**Trial Registration:**

PROSPERO CRD42023456658; https://tinyurl.com/ytnjtmca

## Introduction

### Background

In recent years, the use of mobile health (mHealth) apps by both the public and health care professionals (HCPs) has significantly increased with the introduction of smartphones [1] and growing interest in the health care industry and research field [2]. The COVID-19 pandemic has further accelerated reliance on digital health [3]. mHealth apps are used by patients to manage diseases, self-monitor, gather health information, supervise behavior changes, manage fitness, and remind them of their medication and rehabilitation schedules [4]. From HCPs’ point of view, mHealth apps help manage health records, provide easy access to health records, and provide a path to conduct mobile consultations and remote monitoring during and after treatment [5]. In addition, mHealth apps provide easy access to HCPs by connecting them to clinical information system resources such as electronic health records [6]. Although mHealth apps could provide evidence-based and cost-effective health data and 2-way communication between patients and their HCPs in a remote setting, a few barriers have blocked the expansion of mHealth apps in the health care industry. Data confidentiality, privacy, and security and the regulatory supervision of the apps are some known barriers that hinder mHealth adoption in the health care field.

Despite various benefits of mHealth apps, data confidentiality, privacy, and security issues have caused patients or the public to display less interest and low confidence in mHealth app practice [7]. It could be due to the uncertainty about the information gathered or kept in mHealth apps, the function of the stored data, and who can view or access the data [4]. The term “confidentiality” is defined as the responsibility of those who obtain data (app providers) to uphold the concerns of those to whom the information is related (consumers) [8]. The study by Bhuyan et al [9] mentioned that the National Committee on Vital and Health Statistics defined privacy as an individual legal right or freedom to protect or disclose their health information, and security is defined as personal, mechanical, or authority protection tools used to guard health information against unwanted people or access, whereas privacy is defined as the physical, mechanical, or legislative mechanism or tools to shield personal health information from unauthorized disclosure [10].

Confidentiality, privacy, and security act as challenges in boosting mHealth app adoption. Patients’ perceptions of these issues may influence their adoption of mHealth apps, but such events are context dependent. While users are more likely to raise concerns about privacy or confidentiality issues when probed about mHealth apps, such perceptions may not ultimately influence their behavior regarding the actual use or adoption of such apps. Thus, it is pertinent to explore whether privacy concerns are prioritized by users when they engage with mHealth apps and whether such concerns affect their decision to use the apps or not.

There are several reasons for data protection in mHealth apps, particularly to address the risk of any unauthorized to keyed-in information and stored data by hackers [9]. In addition, data management and storage, data privacy disclosure, data integration, data encryption, app operability, and authentication are established factors contributing to data breaches [9].

Several studies have highlighted the connection between patients’ awareness and the risk of data breaches. End users have an obligation for the security and privacy of their data to be maintained [4]. As the main stakeholders of the health care system, patients have a contractual relationship with health care providers as the latter are expected to ensure the safety and confidentiality of patients’ health information. Health care app developers must protect sensitive patient data by complying with data privacy regulations such as the General Data Protection Regulation (GDPR) and HIPAA (Health Insurance Portability and Accountability Act). To ensure data privacy and security, mHealth apps are encouraged to incorporate data encryption, implement secure authentications, and perform regular risk assessments [10]. While HIPAA encompasses physical, administrative, and technical aspects to ensure the security of personal health information, the GDPR requires health centers or organizations to collect detailed consent from users before recording their personal data and giving them the right to access, amend, delete, or restrict the processing of their data. These privacy requirements for app security are known not only by mHealth app providers but also by patients and users [10,11]. This represents another dimension that may influence patients’ perspectives on and adoption of mHealth apps.

### Objectives

Despite the pivotal role of patients’ views and awareness in the successful implementation of mHealth apps, as demonstrated in several studies, the findings are yet to be summarized to elucidate the barriers and facilitators, which may assist clinicians, HCPs, policy makers, and other stakeholders in their decision-making processes. A previous systematic review on the security and privacy of mHealth apps was conducted almost a decade ago and did not focus on any specific stakeholders [12]. Meanwhile, 2 other reviews related to this topic were a narrative and a scoping review [13,14], which are open to bias as the methods used in retrieving the reviewed articles were not succinctly described. This study aimed to fill the research gap by conducting a systematic review to elucidate patients’ perspectives and awareness of the privacy, security, and confidentiality of mHealth apps, as well as the associated factors.

## Methods

This study was conducted using the PRISMA (Preferred Reporting Items for Systematic Reviews and Meta-Analyses) guidelines [15]. This systematic review was retrospectively registered in PROSPERO (CRD42023456658).

### Search Strategy

The systematic article search was conducted using 3 electronic databases: PubMed, PsycNet, and ScienceDirect. PsycNet was accessed via Ovid as a search interface. These databases were selected given their suitability and specificity for research in health and medical sciences, thus increasing the chances of retrieving articles relevant to the research topic. The first author of this systematic review performed the literature search from February 2022 to April 2022. Articles published between 2014 and 2022 and written in English were considered in the literature search. We focused on studies published from 2014 to 2022 given the growing interest in the use of mHealth apps in the last decade [2].

Aligning with the objectives of this review, the search terms were broadly categorized into 3 components or groups of keywords. Alternative keywords were permitted for each component as denoted using the Boolean operator “OR.” The separator “AND” was then used to combine each component with other wordings. The search query for each of the databases is presented in Table 1.

**Table 1:** The search terms used and the total number of publications retrieved from each database.

Database	Search string and search terms	Initial search results (number of articles)	Date of retrieval
Scopus	Main search terms using document title and abstract: (“Mobile health” OR mhealth OR “mobile phone*” OR “Smart phone*” OR Smartphone* OR tablet*) AND (“Perspective OR opinion* OR attitude* OR perception* OR awareness”) AND (Privacy OR confidential* OR security)	1277	February 20, 2023
ScienceDirect	(“Mobile health” OR mhealth OR “mobile phone”) AND (“Perspective OR opinion OR awareness”) AND (Privacy OR confidential OR security)	7156	March 21, 2023
APA PsycNet	“Mobile health” OR mhealth OR “mobile phone*” OR “Smart phone*” OR Smartphone* OR tablet* AND “Perspective OR opinion* OR attitude* OR perception* OR awareness AND Privacy OR confidential* OR security”	441	April 19, 2023

### Study Eligibility Criteria

The inclusion and exclusion criteria of the review are presented in Table 2. The first inclusion criterion was the document type, whereby only published original articles were considered. Other document types, such as review articles, chapters in books, books, and conference proceedings, were all excluded. The next inclusion criterion was the publication year, whereby only articles published from 2014 to 2022 were selected. The inclusion or exclusion of retrieved articles was based on agreement among the authors. Issues arising during the process were resolved through consensus.

**Table 2:** Inclusion and exclusion criteria.

Consideration factor	Inclusion criteria	Exclusion criteria
Study design	Empirical studies involving qualitative, quantitative, or mixed methods	Reviews (systematic, scoping, narrative, and rapid reviews and meta-analyses)
Publication type	Peer-reviewed journal articles	Editorial letters, protocols, expert opinions, policy briefs, theses or dissertations, and conference papers
mHealth^a^ apps	Studies involving mHealth apps regardless of their aim, target disease, and system type (eg, iOS or Android)	Studies involving apps that are not linked to CISs^b^
Study participants	Studies involving patients with or without HCPs^c^, such as physicians, nurses, pharmacists, and care teams, regardless of demographic characteristics (ie, age, gender, and ethnicity)	Studies solely involving app developers, HCPs, and telehealth providers
Outcomes	Experience, perceptions, awareness, and knowledge of patients after practical use of mHealth apps	Description of the impact of mHealth apps on the patient-HCP relationship
Language	English	Other languages
Year	2014 onward	Before 2014

^a^mHealth: mobile health.

^b^CIS: clinical information system.

^c^HCP: health care professional.

### Study Selection

In total, 2 researchers performed the study selection independently. Articles retrieved from the primary literature search from each database were sent to the researchers’ email repositories and stored for future reference. The screening process was carried out using the filter feature available in all 3 databases. The initial search results were checked for duplicates, which were then identified and removed accordingly. The last screening stage was full-text reading.

### Data Extraction

The final articles included in this study were assessed, reviewed, and examined upon completing the eligibility process. An Excel (Microsoft Corp) spreadsheet form was created to use in data extraction. The data extracted from the studies were as follows: first author; year of publication; study location; study design; types and purposes of mHealth apps; issues related to the privacy, security, and confidentiality of mHealth apps; and the main findings. Data extraction was performed by the first author, and thus, the intercoder agreement was not assessed.

### Quality Appraisal

Quality appraisal was not performed in the review due to the heterogeneity of the research objectives, designs, and methodology used in the included studies [16].

### Data Synthesis

A narrative synthesis was considered in this review due to the heterogeneity of the designs used in the studies. Specifically, thematic analysis was conducted to summarize the findings of the included studies. The data extracted were analyzed thematically. All the authors participated in the discussion to determine the themes that would be synthesized from the analysis. The themes decided on were (1) barriers to and facilitators of using mHealth apps and (2) recommendations to increase the use of mHealth apps by addressing privacy, security, and confidentiality issues. Any further analysis and reassessment of the themes and subthemes was conducted continuously.

## Results

### Search Outcomes

**Figure 1:**
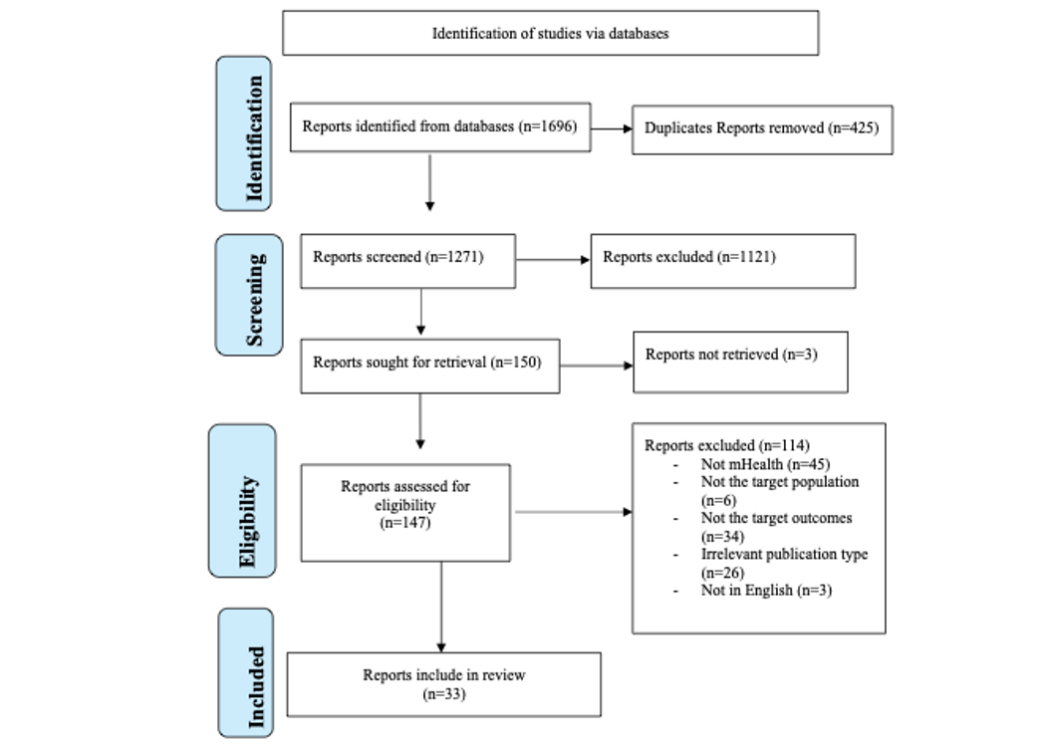
PRISMA (Preferred Reporting Items for Systematic Reviews and Meta-Analyses) flow diagram of the study selection process.

A total of 1696 articles were retrieved from the initial searches on PubMed (n=659, 38.86%), ScienceDirect (n=172, 10.14%), and Ovid (n=865, 51%). Of the 1696 search results, 425 (25.06%) were removed from the list as duplicates (Figure 1), whereas 1121 (66.1%) were considered ineligible upon screening the titles and abstracts. The 150 remaining articles were then subjected to a full-text review, which led to the final selection of 33 (22%) articles for the systematic review based on the inclusion and exclusion criteria (Figure 1).

### Characteristics of the Studies

As shown in Table 3, the highest number of studies was published in 2021 (9/33, 27%) and 2019 (7/33, 21%). The included studies were quantitative (13/33, 39%), qualitative (11/33, 33%), and mixed methods (9/33, 27%). In terms of study location, most of the studies were conducted in countries with countries with sufficient resources (26/33, 79%) compared to those undertaken in resource-limited countries (7/33, 21%). While 9% (3/33) of the articles emphasized the general use of mHealth apps for routine health records, 36% (12/33) of the articles focused on specific mHealth apps for collecting patients’ health data and management of various medical conditions, such as chronic obstructive pulmonary disease [17], cancer [18-21], and diabetes [22], and pregnancy care [23,24] (Table 4).

**Table 3:** Descriptive information of the articles (N=33).

Variables			Studies, n (%)
**Year of publication**			
	2014	2 (6)	
	2015	2 (6)	
	2016	2 (6)	
	2017	2 (6)	
	2018	4 (12)	
	2019	7 (21)	
	2020	4 (12)	
	2021	9 (27)	
	2022	1 (3)	
**Study design**			
	Qualitative	13 (39)	
	Quantitative	11 (33)	
	Mixed methods	9 (27)	
**Study location**			
	High-income countries	26 (79)	
	Middle-high–income countries	7 (21)	
	Low-income countries	0 (0)	
**Purpose of mHealth^a^ apps**			
	Routine electronic health records	3 (9)	
	Specific apps for patient management	12 (36)	
	Unspecific	18 (55)	

^a^mHealth: mobile health.

**Table 4:** Details of author name, publication year, type of intervention, study design, setting, instruments, and findings of the 33 reviewed articles.

Study	Type of intervention	Study design	Study location	Data collection method	Main findings	Themes identified
Wyatt et al [25]	PhotoExam apps (EHRs^a^)	Quantitative	United States	App use and survey	(1) Only 3% (2/71) of patients expressed privacy or confidentiality concerns related to the photos taken; (2) 26% (18/70) of patients mentioned that the security features of the apps were explained by the HCPs^b^; and (3) 16% (11/70) indicated that the security features were not explained, and most of them (41/70, 59%) did not remember and were unsure of whether the security features were explained	Concerns about privacy; concerns about confidentiality
Zhou et al [4]	mHealth^c^ apps	Mixed methods	United States	Questionnaire and qualitative analysis (semistructured interview and psychometric analysis)	(1) Participants had some level of concern about the privacy of their personal data and wanted to have some specific protections; (2) Participants believed that a level of privacy protection is currently available in mHealth apps; (3) Participants desired to have informed consent, access control, a privacy policy, and remote wiping features in mHealth apps; (4) two-thirds of the users (66.7%) stated that the cost and lack of security features of mHealth apps were the main barriers to adopting the technology.	Concerns about data privacy; concerns about data security
Natsiavas et al [26]	eHealth data	Quantitative	14 European countries	Questionnaire	(1) 11.96% did not think about possible health data risks, and 36.41% felt informed about these risks; (2) 66.21% of the respondents did not read the “Terms and Conditions,” with >30% declaring that they did not feel that it was worth it given the time required to read them and 19.79% declaring indifference toward them; (3) 26.09% of the respondents felt confident regarding their eHealth data privacy, 38.04% felt concerned but helpless, and 16.3% stated that they avoided using eHealth services due to the lack of confidence regarding their data handling; (4) 20% of the respondents felt that their privacy was fully covered in the “Terms and Conditions” of the apps, and 12.5% declared that they did not understand them; (5) 72.46% of respondents were willing to share their personal data for research purposes, at least under anonymization	Awareness of data privacy and confidentiality; concerns about data privacy
Alaiad et al [27]	mHealth model	Quantitative	Jordan	Questionnaire	(1) Security and privacy risks have a direct negative effect on the patients’ intention to use mHealth; (2) mHealth patients in lower-income countries are often using mHealth services at their own risk, which makes them prone to data breaches and misuse by unknown parties	Data security; data privacy
Özkan et al [28]	EHRs	Quantitative	Turkey	Survey	(1) Most of the participants (60.9%) stated that they did not know who had the right to access their medical records, and 7.4% believed to have comprehensive knowledge on the topic; (2) the medical records of 9.7% of the respondents had been used or released without their consent; (3) 15.1% stated that they avoided being tested due to violation risks, and 3.5% asked their physicians to enter a less embarrassing health status in their records; (4) most participants (94%) responded that they should have full access to their medical data, whereas 50% of respondents wanted other parties (children, parents, physicians, spouses, and other hospital staff) to have limited access rights	Awareness of data confidentiality and privacy; data confidentiality; data privacy
Glauser et al [29]	NeuroPath (mHealth app)	Qualitative	United States	App use and telephone interview	14 (63.6%) respondents stated that they waited before trying new technology, and 6 (27.3%) of these respondents were concerned about data security when asked about their readiness to adopt new technologies.	Concerns about data security
Zhou et al [30]	SecSim (security simulator)	Mixed methods, quasi-experimental study and interview session	United States	App use, IBM PSSUQ^d^, and open-ended interview questions	(1) The comparison between the pre– and post–security education selection regarding security settings indicated that 21% (14/66) to 32% (21/66) of participants chose a stronger security measure in text encryption, access control, and image encryption; 0% (0/66) to 2% (1/66) of participants chose a weaker measure in these 3 security features; and the remainder kept their original selections; (2) a significant percentage of patients (21%-32%) needed guidance to make an informed selection regarding security settings	Concerns about data security
Barutçu et al [31]	e-Pulse (mHealth app)	Quantitative	Turkey	Survey	(1) Participants considered mHealth apps as less credible in protecting their personal information; (2) positive relationships were found between user satisfaction level with mHealth apps and ease of use, trust, privacy, usefulness, and information quality of mHealth apps; (3) mHealth apps’ user satisfaction was negatively influenced by the privacy of mHealth apps; (4) perceived ease of use, trust, privacy, perceived usefulness, and information quality were the major factors influencing satisfaction with and intention to adopt mHealth apps	Concerns about data privacy; associated factors: satisfaction with mHealth apps
Bauer et al [32]	mHealth platform supporting collaborative care	Mixed methods	United States	App use, surveys, and interviews	(1) Patients felt that the data they submitted were not entirely secure, but they did not believe that the information reported in the apps was highly personal, and therefore, the potential for a data breach was not a major concern; (2) some patients wished to have a better understanding of who else had access to their health information and the ability to control such access (based on qualitative findings)	Data security; awareness of data security and privacy
Goetz et al [33]	PRELAX (eHealth application)	Mixed methods	Germany	Application use, self-administered questionnaire, and semistructured interviews	(1) Hospitalized women (6/30, 20%) were worried about unauthorized third-party access to their stored medical data; (2) several women (8/30, 27%) expressed concerns about data security, especially in the field of mobile apps as many free apps make private data easily accessible; (3) data security and personal data storage in pregnancy apps were general causes for concern	Data confidentiality; data privacy
Richardson and Ancker [34]	her	Quantitative	United States	Survey	(1) Approximately three-quarters of individuals believed that storing medical information on a phone would threaten privacy and security (74% in 2013 and 75% in 2014); (2) approximately two-thirds thought that sharing data between a mobile device and a physicianherEHR would threaten privacy and security (69% in 2013 and 67% in 2014); (3) participants expressed greater concern about worsened privacy and security with storing data on mobile phones and mobile herne–EHR communication (74% in 2013 and 69% in 2014) than with the health information exchange between EHRs and physicians (41% in 2013 and 47% in 2014)	Data privacy; data security
Sanger et al [35]	Postacute care apps	Qualitative	United States	Semistructured interviews	Participants were most concerned about the collection and transmission of particularly sensitive information, such as photos of the groin area	Data privacy; data security
Dang et al [36]	Not specific	Quantitative survey	China	Survey	Privacy concerns among patients were positively enhanced by perceived health information sensitivity (β path coefficient=0.505; *P*<.001). Patients’ health information disclosure intention decreased significantly with higher concern levels (β path coefficient=–0.338; *P*<.001). The relationship between perceived health information sensitivity and privacy concerns was negatively moderated (β path coefficient=–0.17; *P*=.09) by the informational support dimension. A similar moderating effect was observed in the association between privacy concerns and health information disclosure intention (β path coefficient=–0.11; *P*=.09).	Data privacy; data security; associated factors: health information disclosure
Alwashmi et al [17]	Not specific	Mixed methods	Canada	In-depth interviews and survey instrument	The barriers to adoption were technical issues, lack of awareness, potentially limited uptake from older adults, and privacy and confidentiality issues.	Awareness of data privacy and confidentiality; associated factors: demographic factors; data privacy issues and confidentiality issues; facilitators and enablers
Biswas et al [37]	ACCU3RATE (a specific AI^e^-enabled mHealth app rating scale)	Quantitative	Multinational study	Survey instrument	Patients provided positive feedback regarding the apps’ features for accessibility, protection, and privacy of patient data.	Data privacy
Casilang et al [38]	mHealth for the development of an exclusive breastfeeding tool	Qualitative	Dominican Republic	Focus group discussion	Barriers to mHealth use included the cost of internet service, privacy concerns, and perceived credibility of information sources	Facilitators and enablers; data privacy
Zhang et al [39]	Unspecific	Quantitative	United States	Web-based survey	Data type (*P*=.003), data stage (*P*<.001), privacy victimization experience (*P*=.01), and privacy awareness (*P*=.08) showed positive effects on patients’ privacy concerns. Higher privacy concerns were reported for social interaction data (*P*=.007) and self-reported data (*P*=.001) than for biometric data. Privacy concerns were also higher for data transmission (*P*=.01) and data sharing (*P*<.001) than for data collection. Privacy concerns affected the attitude toward privacy protection (*P*=.001), thereby affecting continuous use intention.	Privacy concerns; associated factors: data type and stage and victimization experience
Harris et al [40]	Mobile phone health apps	Mixed methods	Pakistan, Tanzania, Kenya, Nigeria, and Bangladesh	Scoping study and analysis of survey data	Stakeholder willingness was high provided challenges regarding technology, infrastructure, data security, confidentiality, acceptability, and health system integration were addressed. Mobile consultations can reduce affordability barriers and facilitate care-seeking practices.	Data security; data confidentiality
Moodley et al [18]	Mobile phone health apps	Mixed methods	South Africa	Survey and focus group discussion	Users were interested in the use of mobile phone apps for health intervention in receiving Papanicolau smear results and appointment reminders. However, concerns were raised regarding the confidentiality of SMS text messages, loss or theft of mobile phones, receiving negative results, and the accessibility or clarity of the language used to convey the messages.	Data confidentiality
Li et al [24]	mHealth technology for monitoring pregnancy care	Qualitative	Australia	In-depth interviews	The clinical and technical challenges regarding the introduction of mHealth for pregnancy care were also identified, whereas usability and data privacy were among the main concerns of the participants.	Data privacy; facilitators and enablers
Gill et al [21]	mHealth technology to support postabortion care	Mixed methods	United States	Survey and semistructured interviews	Qualitative analysis revealed that participants preferred a comprehensive website with secured email or SMS text message notifications to provide tools and resources for emotional well-being, contraceptive decision-making, general sexual health, and postprocedural care.	Data security; facilitators and enablers
Bradbury et al [19]	Remote real-time videoconferencing for patients with cancer	Quantitative and experimental	United States	Experimental design	Most patients reported that their privacy was respected after the first (56/57, 98%) and second sessions (40/41, 98%), respectively. Meanwhile, some patients reported concerns that RVC^f^ might increase the risk of a confidentiality breach of their health information—after V1: 14/57 (25%); after V2: 12/41 (29%).	Data confidentiality
Al-Anezi [41]	Unspecific	Quantitative	Saudi Arabia	Survey	Fear of privacy violations, fear of loss of personal data and information, and lack of technical support were highlighted as the main reasons for the lack of motivation to adopt the mHealth system	Privacy issues
Ermakova et al [42]	Health clouds	Quantitative	Germany	Survey	Confidentiality assurance was vital in influencing individuals’ acceptance of health clouds for sensitive medical data, but such an effect was lacking for nonsensitive medical data.	Data confidentiality
Rodrigues et al [43]	Unspecific	Qualitative	South India	In-depth interviews	Participants perceived the risk of unintentional disclosure of their HIV status and the stigma thereof via the intervention and took initiatives to mitigate this risk.	Data confidentiality
de Vries et al [22]	Unspecific	Qualitative	The Netherlands	Focus group discussions and interviews	The use of mobile apps for reporting ADRs^g^ was influenced by source of information, app’s security, type of feedback, storage pattern of ADR reports, ease of use, and the type of language.	Data security; facilitators and enablers
Al-Mahrouqi et al [44]	Unspecific	Qualitative	Oman	Semistructured qualitative interviews	Although clients acknowledged the positive impact of telehealth in improving mental health care services in Oman, primary concerns were related to privacy, the security of telehealth systems, lack of public tele–mental health services, lack of specified tele–mental health guidelines, shortage of trained therapists, and limited access to high-speed internet and electronic devices.	Concerns about data privacy; concerns about data security
Turcotte et al [45]	Teleconsultation (AYP^h^)	Qualitative	Canada	Semistructured interviews	Although both users and nonusers showed positive experiences and perspectives on the AYP platform were mostly positive, concerns were raised regarding patients’ safety.	Data security issues
Hackett et al [23]	Mobile phone apps	Qualitative	Tanzania	Semistructured interviews	(1) Perceptions of personal privacy and confidentiality were negatively and positively impacted by the use of new technologies to capture health service user data during pregnancy and childbirth; (2) women’s concerns regarding privacy aligned closely with a belief that pregnancies and expected delivery dates must be kept secret, reflecting fears that pregnancy renders women vulnerable to witchcraft by jealous neighbors; (3) they were also concerned that health workers’ male partners could access their private information.	Concerns about data privacy; concerns about data confidentiality; facilitators and enablers
Morton et al [46]	Mobile phone apps	Mixed methods	Many countries	Survey and in-depth interviews	Concerns regarding data security were prevalent. Data security, content quality or accuracy, ease of use, and cost were among the prioritized mHealth features. The ability to share data with others was described as vital by less than half of the respondents.	Concerns about data security
Cavazos-Rehg et al [47]	mHealth mental health intervention	Quantitative	United States	Survey	The main reasons provided for unwillingness to obtain parental consent to participate in the intervention included the importance of preserving privacy and the feeling that parents lack awareness or understanding of mental health issues.	Privacy concerns; awareness of data privacy
Sangers et al [20]	mHealth apps for skincare screening	Qualitative	The Netherlands	Semistructured interviews	The main barriers to using mHealth apps included privacy concerns, perceived lack of value, perception of untrustworthiness, preference for a physician, a complex user interface, and high costs.	Privacy concerns; facilitators and enablers
Hattingh et al [48]	mHealth in pharmacy settings	Qualitative	Australia	In-depth interviews and focus group discussion	Consumers indicated a desire to receive information in a way that respects their privacy and confidentiality in an appropriate space. Important areas were identified that require improved protection of privacy and confidentiality during pharmacy interactions.	Privacy, security, and confidentiality issues

^a^EHR: electronic health record.

^b^HCP: health care professional.

^c^mHealth: mobile health.

^d^PSSUQ: Post-Study System Usability Questionnaire.

^e^AI: artificial intelligence.

^f^RVC: remote videoconferencing.

^g^ADR: adverse drug reaction.

^h^AYP: Ask Your Pharmacy.

### mHealth Apps for Specific Interventions

A total of 36% (12/33) of the studies included in this review reported the use of mHealth apps for patient health data collection and management of specific health conditions. In total, 9% (3/33) of the studies entailed the use of specific mHealth apps for assessing patient treatment progress [25,29,35]. PhotoExam apps [25] entailed the collection of patients’ photos, which were then assessed for patient response to the treatment provided. Glauser et al [29] developed an app named “NeuroPath” with the support of Apple, the Institutional Department of Neurosurgery, and the Department of Information Technology. The areas targeted by the app included patient surgical preparation, prevention of perioperative risk, wound care, activity monitoring, and opioid use management. Meanwhile, Sanger et al [35] focused on mHealth apps for postacute care.

Regarding specific medical conditions, 9% (3/33) of the studies focused on mHealth apps for pregnancy management, such as a patient engagement pregnancy app (PRELAX) [33], pregnancy care [24], and support for postabortion care [14]. Other studies involved mHealth apps for real-time videoconferencing for patients with cancer [19], mental health interventions [47], support for collaborative care [32], and teleconsultation for pharmaceutical services [43].

Finally, 6% (2/33) of the studies emphasized the security of mHealth apps without focusing on medical conditions or groups of patients. Zhou et al [30] developed a security simulator named SecSim to reveal the consequences of selecting different security options available in the security settings of mHealth apps. Meanwhile, Biswas et al [37] used a specific artificial intelligence–enabled mHealth app rating scale, ACCU3RATE, to obtain users’ feedback on the security features. The influence of these interventions on patient perception on the security, confidentiality, and privacy of data collected via mHealth apps is presented in the thematic analysis.

### Results of the Thematic Analysis

The thematic analysis generated four broad themes from the findings of the studies: (1) privacy, confidentiality, and security; (2) awareness of privacy, security, and confidentiality; (3) facilitators and enablers; and (4) associated factors. The following subsections present more detailed information about the synthesized themes.

#### Theme 1: Privacy, Confidentiality, and Security

As expected, all the included studies (33/33, 100%) investigated privacy, confidentiality, and security issues related to patients’ use of mHealth. Different levels of privacy and confidentiality concerns were reflected in the studies. In 15% (5/33) of the studies, less than half of the patients expressed concerns about the privacy or confidentiality of the various data required by mHealth apps [19,25,26,28,33]. Meanwhile, in 6% (2/33) of the studies, >50% of the respondents raised diverse issues regarding the privacy and confidentiality of their data [4,34]. In terms of data security, 9% (3/33) of the studies, which used a quantitative design, revealed that a higher proportion of patients (>50%) acknowledged issues related to data security [29,30,34].

This theme was also depicted in several qualitative and mixed methods studies [4,20,22-24,37,39,40,45,48]. For instance, patients opined that the privacy protection level in mHealth apps needed to be improved [4,29,38]. Meanwhile, patients felt that their data were not completely secure and were concerned about data breaches [21,32]. In 9% (3/33) of the studies, issues related to privacy, confidentiality, and security were identified as barriers to mHealth use [24,38,40]. Meanwhile, Biswas et al [37] found that respondents were satisfied with the apps’ features for the protection and privacy of patient data.

#### Theme 2: Awareness of Privacy, Security, and Confidentiality

Patients’ awareness of mHealth apps’ privacy, security, and confidentiality was highlighted in 12% (4/33) of the studies [4,25,26,28]. Nevertheless, the awareness level differed across the studies.

Natsiavas et al [26] found that 12% of patients in their study were unaware of the possibility of health data risks, whereas a higher percentage of participants (61%) in the study by Özkan et al [28] did not know who had the right to access their medical records. Thematic analysis of the data gathered by Bauer et al [32] revealed that patients opted for a better understanding of other parties who have access to their health information and their capacity to regulate such access. Meanwhile, Alwashmi et al [17] and Zhang et al [39] identified awareness of privacy and confidentiality issues as barriers to adopting mHealth apps and raised concerns about data privacy, respectively.

#### Theme 3: Facilitators and Enablers

The third theme synthesized in this systematic literature review is the facilitators of increased use or adoption of mHealth apps among patients based on the perceived benefits of mHealth apps and recommendations to address data privacy, security, and confidentiality issues. In total, 18% (6/33) of the studies reported the perceived benefits of mHealth apps that may reduce patients’ concerns about issues related to data privacy and security, thus improving the adoption rate of such apps [17,21-24,30]. Some of the benefits highlighted by patients included improved health status by reducing the rate of hospitalization [17], increased trust, better patient-HCP relationships [22,23], and exchange of information in real time [21]. Overall, patients viewed mHealth apps installed on smartphones as an added value, which assisted in improving the confidentiality of their data, their trust, and their relationship with health care personnel.

As for recommendations and facilitating conditions to address data privacy, security, and confidentiality issues related to mHealth, the consistent points raised in the studies were the protection of personal data, ensuring that health status or medical conditions are not mentioned, brief training or education on data security, and assuring data confidentiality and privacy [21,24,30]. Gill et al [21] found that participants prioritized privacy and confidentiality by preferring discrete mHealth designs that did not mention the specific medical condition that prompted them to visit the clinic.

#### Theme 4: Associated Factors

The last theme gleaned from this review was the factors associated with patients’ concerns regarding issues related to data confidentiality, privacy, and security when using mHealth apps. This theme was synthesized from the findings reported in 15% (5/33) of the articles [4,31,36,39,42], which comprised patients’ sociodemographic factors, satisfaction with mHealth, data type and stage, and experience with mHealth apps.

In terms of sociodemographic factors, Zhou et al [4] found that married patients showed higher information security and privacy concerns and desired more stringent security protection compared to single patients. The weakest concerns about privacy and security were exhibited by users with <US $10,000 in annual income compared to patients who earned >US $75,000 annually. Similarly, patients in the older age group (51-65 years) reflected a higher level of concern about privacy in mHealth apps relative to the younger age group (18-28 years). In terms of experience, participants who had previously used mHealth apps had greater concerns about data security and privacy despite still being interested in continuing to use the technology.

Only 3% (1/33) of the studies reported the association between satisfaction levels with mHealth apps and privacy concerns [31]. Specifically, a positive relationship was observed between user satisfaction levels with mHealth apps and privacy concerns. Meanwhile, Dang et al [36] found that a higher perceived health information sensitivity heightened the privacy concerns (β path coefficient=0.505; *P*<.001) raised by patients regarding mHealth apps. The provision of informational support moderated the association between privacy concerns and health information sensitivity.

Zhang et al [39] revealed the diverse levels of privacy concerns depending on data type, data stage, and privacy victimization experience. For instance, privacy concerns were higher for patients’ social interaction, self-reported, and biometric data. Users were less concerned about privacy issues during data collection compared to the data transmission and sharing stages. These events had negative impacts on the continuous intention to use mHealth apps [39]. Ermakova et al [42] also found that patients’ acceptance of health clouds for nonsensitive medical data was not significantly affected by confidentiality assurance; however, this relationship was significant for sensitive medical data.

## Discussion

### Principal Findings

This systematic review evaluated patients’ perspectives and understanding of the data confidentiality, privacy, and security of mHealth apps connected to clinical information systems. A total of 33 relevant articles were extracted and included in this systematic review using the PRISMA guidelines. Descriptive analyses revealed that most of the studies (26/33, 79%) were conducted in high-income countries compared to those undertaken in middle- to low-income countries (7/33, 21%). These findings reflect the disparity in the implementation and adoption of mHealth apps in line with different countries’ economic status and infrastructural capacity. This is evident in the use of mHealth apps for the management of specific medical conditions in the United States, the United Kingdom, Australia, and Turkey, whereas the few studies conducted in lower-income countries focused mainly on either the feasibility or introductory stages of general mHealth apps. Notwithstanding the patients’ socioeconomic status, data privacy, confidentiality, and security issues were highlighted in most studies.

The extensive research on mHealth apps was also reflected in the diverse medical conditions in which the technology was explored, such as chronic obstructive pulmonary disease [17], cancer [19,20], postabortion care [21], diabetes [22], and pregnancy care [24]. Descriptive analyses also revealed the use of various research methods (qualitative, quantitative, and mixed methods) in the reviewed articles, which is not surprising given that the research topic can be explored via interviews, focus group discussions, and surveys.

Thematic analyses of the qualitative studies revealed 4 main themes, comprising primary concerns about privacy, confidentiality, and security of data; awareness of privacy, security, and confidentiality issues; facilitators and enablers; and associated factors. Regarding the first theme, patients were concerned about data security and privacy, particularly in terms of the collection and transmission of sensitive information such as identity-revealing data and images of body parts [22,33,35]. Some hospitalized patients were even more concerned about unauthorized third-party access to their medical data given that mHealth apps are mostly free and easily accessible [17,33]. In contrast, some patients were indifferent to these issues in the same studies as they were willing to use mHealth apps and share their health data with HCPs. These diverse views could be linked to patients’ consideration of the benefits and risks associated with using mHealth apps for routine health records or managing their health conditions.

We observed concordant perspectives on the research topic as patients and end users consistently raised concerns about data privacy, security, and confidentiality issues that prevented them from using mHealth apps [18,20,35]. This result aligns with those of a previous review by Nurgalieva et al [13] in which low levels of security and privacy were reported as the main reason for low use among patients and end users. Serious issues may arise from mHealth apps with low levels of security or privacy, and such events may have severe consequences for users and organizations.

Given the extensive privacy and security issues raised in the reviewed studies, the findings suggest the need for mHealth environments to improve the security of these apps by exploring advances in cyberspace security [4]. Similarly, the reviews both by Martínez-Pérez et al [14] and Nurgalieva et al [13] highlighted security incidents, including vulnerabilities discovered in widely used mHealth apps and malware attacks. The concerns raised by patients are plausible as the proliferation of mobile devices with location sensors has facilitated access to location-based services [14]. These advanced devices transmit the user’s location information to third-party location servers, which are accessible by other service providers. Users aware of this potential data breach may feel that they are continuously tracked. In this review, some patients seemed to be aware of the risk of third-party and unauthorized access to their medical data [4,25].

For the second theme, both qualitative and empirical findings reflected that patients’ awareness of the privacy and security issues of mHealth apps differed across the studies. For example, more than two-thirds of participants expressed their concerns about personal data security and privacy and requested user authentication and data encryption to protect user data [4,34], and <12% were unaware of the risk of health data breaches [19,31]. Meanwhile, more than half of the respondents in the studies conducted by Özkan et al [28] and Wyatt et al [25] were unsure about the privacy and security information on their mHealth apps. Thus, the different awareness levels among patients may influence their diverse perspectives on the privacy and security of mHealth apps.

The third theme emerging from the thematic analyses entailed the facilitators for increased use or adoption of mHealth apps based on patients’ perceived benefits of mHealth apps and suggestions to address data privacy, security, and confidentiality issues. Resultantly, improved health status [17], better patient-HCP relationships [22,23], and trust were the main benefits mentioned by patients. On the other hand, personal data protection, ensuring that health status or medical conditions are not mentioned, brief training or education on data security, and assuring data confidentiality and privacy were the consistent recommendations provided by patients [21,24,30]. These findings coincide with the suggestion by Perera [49] regarding the use of an alphanumeric passcode to ensure the protection of mHealth apps rather than using a 4-digit personal identification number, as well as wiping data from the mobile device after a specific number of failed passcode attempts [49,50]. In their review, Nurgalieva et al [13] also emphasized the frequency of notifications and alerts programmed into mHealth apps. Accordingly, discreet or private notifications were advised to prevent any distress to users, particularly in situations in which someone else could accidentally view the app icon. Overall, these recommendations reflect the need for users to have complete control in using their mobile devices for mHealth and avoid any intrusion in their daily life. Training and education as recommended by patients in this review corroborate the report by Lewis and Wyatt [50] as reviewed by Nurgalieva et al [13]. The latter authors suggested that the risk factors for violation of users’ privacy and security in mHealth apps can be categorized into external and internal risks. Appropriate regulation can be used to effectively minimize the internal risk factors, whereas proper education and training are pertinent to eliminate the external risk factors.

Concerning the fourth theme, information on the factors influencing patients’ views regarding data privacy and confidentiality was obtained from a few of the studies included in this systematic review (5/33, 15%). Examples included sociodemographic characteristics such as age, income level, marital status, previous experience with mHealth apps [30], patients’ satisfaction levels [31], perceived health information sensitivity [36,42], data type, data stage, and privacy victimization experience [39,42]. These findings are consistent with the results of several previous studies [51] and the contextual nature of the theory of privacy [52]. More importantly, certain adjustments to the security and privacy features of mHealth apps need to be incorporated by the app developers upon considering patients’ and users’ demographics. However, given the low number of studies reporting the underlying factors influencing patients’ views on data privacy, confidentiality, and security issues in mHealth apps, more research is needed to elucidate the relationships.

### Implications of the Findings

The findings of this study have pertinent implications for mHealth app developers, HCPs, and policy makers. Both HCPs and mHealth app developers have a vital role to play in addressing the diverse views exhibited by patients and end users on data privacy, confidentiality, and security issues in mHealth apps.

While mHealth app developers are primarily responsible for designing security measures and features to ensure their apps’ data privacy and confidentiality, from the perspective of health care provider and patient relationships, the former play a pivotal role in educating patients or end users regarding such measures. This could be discussed during routine consultations, as well as reiterating the need for collecting sensitive data and what they are going to be used for. Regarding unauthorized third-party access and potential data breaches, users may benefit from information on the privacy requirements and meeting of the standards set by the GDPR and HIPAA, which are designed to ensure that such privacy issues and data breaches are prevented.

Apart from informing patients and end users on the type of data to be collected and the intended use, the aspect of training and educating end users on the available features and measures to ensure data privacy and security cannot be overemphasized. Meanwhile, stakeholders need to gauge the users’ level of awareness and knowledge of these issues as well as the underlying reasons for the diverse views on these issues to tailor educational interventions accordingly. As gleaned from the studies included in this review, users’ lower concern about their data privacy may stem from being completely ignorant, or they may perceive that the benefits of using mHealth outweigh the potential risks.

This study also has important implications for mHealth app developers given the fact that patients and end users have raised concerns about data privacy, security, and confidentiality issues that end up affecting their use of such apps [18,32]. Nevertheless, accumulated findings reflect that patients’ perceptions on privacy issues and the latter’s influence on patients’ adoption of mHealth apps are context dependent [39,43]. As most of the reviewed studies involved surveys, it is expected that users will raise concerns on privacy or confidentiality issues related to mHealth apps, but further inquiries are required to elucidate whether such concerns influence their behavior and actual use of such apps. These events need to be succinctly explored in future studies, and stakeholders need to understand this gray area to effectively address the issues. In addition, mHealth app developers may evaluate the issues raised by users and strategize on how to improve their current security and privacy measures, particularly ensuring that they meet the requirements of the HIPAA and GDPR. Furthermore, the appropriate actions to be taken by end users in different circumstances should be clarified in the developed security policy and guidelines. In summary, providing suitable awareness in the security guidelines and policies for end users is as pertinent as developing secure mHealth apps.

For policy makers and researchers, this study highlights the aspects prioritized by patients regarding the adoption of and desired outcomes of implementing mHealth apps, as well as addressing issues related to data privacy and security. Accordingly, better patient-HCP relationships, trust, personal data protection, ensuring that health status or medical conditions are not mentioned, and brief training or education on data security were the consistent recommendations provided by patients [19,23,24]. While most of the aforementioned points can be conveyed to users, trust is a feature that has to be earned, which has to be driven by health care providers and the government. These stakeholders also have to mitigate apprehensions related to patients’ privacy concerns to ensure enhanced trust between patients and service providers, which is crucial for the successful delivery of eHealth services.

Certain concrete actions can be taken by stakeholders at the government level by considering HIPAA and the European Union Safe Harbor law, which advocates for strict security measures for the exchange and sharing of health data. Failure to comply with such laws entails severe consequences. From health care providers’ perspective, and given the confidentiality and sensitivity of patient data, only authorized users such as medical staff should be given access to stored health data. Nevertheless, confidentiality and availability need to be carefully balanced when structuring this critical security system. Despite the fact that all patients’ health data are made available to be exchanged, shared, and monitored to provide robust health care services, certain aspects of the data may be considered confidential and, for security reasons, must be kept restricted or inaccessible. These goals should be rationalized to ensure that patients receive the best possible care.

### Strengths and Limitations

This study involved a comprehensive systematic literature search and identification of relevant and recent articles on mHealth apps’ privacy, security, and confidentiality published in the last 8 years. Detailed information on patients’ perspectives and awareness of the privacy and security of mHealth apps were gleaned from this review, thus bridging the current research gap as no systematic review has been conducted on this topic. Thematic and empirical analyses were also conducted to obtain robust data from the various designs used in the studies and triangulate the findings.

Nevertheless, the limitations of this study are well acknowledged. The literature search was restricted to 3 databases; thus, some important articles on the research topic might have been missed. Only patients and end users were considered as the primary targets in this review, whereas the perspectives of other active stakeholders in mHealth apps, such as HCPs, app developers, and policy makers, were not documented. The issues raised in this study might be better understood if all relevant stakeholders were considered. This also limits the generalizability of the findings as no inference could be made regarding health care personnel and mHealth app developers. The use of 1 reviewer for data extraction is also an important limitation; however, the reviewer was trained on how to perform the data extraction and coding before the study to ensure that the process was reproducible and consistent.

Meanwhile, a general limitation of the retrieved articles is the need for a clear definition of data privacy and security. Most of the reviewed studies considered security and privacy as a single concept, particularly as part of a general assessment of mHealth app design. Although security and privacy may overlap when ensuring patients’ confidentiality, the 2 concepts are fundamentally different. In addition, only 3% (1/33) of the studies assessed the relationship between patients’ sociodemographic factors and their concerns regarding mHealth apps’ privacy, security, and confidentiality. A more robust assessment of patients’ demographic characteristics, environmental factors, and patients’ antecedents regarding data breaches and leakage to unwanted third parties requires further investigation.

### Conclusions

This systematic review elucidated patients’ perspectives and awareness regarding mHealth apps’ privacy, security, and confidentiality. Patients showed diverse perspectives on the trio of concepts, ranging from users who were satisfied with the privacy and security features of their current mHealth apps to those who raised pertinent issues affecting technology use. Patients also conveyed specific approaches to improve the use of mHealth apps (facilitators), such as protection of personal data, ensuring the confidentiality of health status or medical conditions, and provision of brief training or education on data security and privacy.

The aggregation of the empirical and thematic results reflects that these diverse perspectives might be linked to the awareness of the subject matter, which also differed across the studies and was influenced by patients’ sociodemographic characteristics, such as age, income level, and marital status, as well as their experience with mHealth apps, satisfaction levels, data type, and data stage. Thus, the findings of this review may be beneficial to mHealth app developers and other stakeholders in improving patients’ awareness and adjusting current privacy and security features to enhance the use and adoption of mHealth apps for routine health monitoring and management of specific health conditions.

## Appendix

Multimedia Appendix 1 PRISMA (Preferred Reporting Items for Systematic Reviews and Meta-Analyses) checklist.

## Abbreviations

GDPR: General Data Protection Regulation
HCP: health care professional
HIPAA: Health Insurance Portability and Accountability Act
mHealth: mobile health
PRISMA: Preferred Reporting Items for Systematic Reviews and Meta-Analyses

## Data Availability

The data sets generated during and analyzed during this study are available from the corresponding author on reasonable request
